# Multi-Strain Probiotic Lysate Attenuates TGF-β1-Induced Intestinal Fibrosis and EMT Modulating Smad, Akt, and WNT/β-Catenin Pathways

**DOI:** 10.3390/cells14181432

**Published:** 2025-09-12

**Authors:** Alessia Ciafarone, Serena Artone, Valeria Ciummo, Francesca Rosaria Augello, Serena Altamura, Francesca Lombardi, Giovanni Latella, Paola Palumbo, Benedetta Cinque

**Affiliations:** 1 Department of Life, Health & Environmental Sciences, University of L’Aquila, 67100 L’Aquila, Italy; alessia.ciafarone@univaq.it (A.C.); serena.artone@univaq.it (S.A.); francescarosaria.augello@univaq.it (F.R.A.); serena.altamura@univaq.it (S.A.); francesca.lombardi@univaq.it (F.L.); giovanni.latella@univaq.it (G.L.); paola.palumbo@univaq.it (P.P.); 2 Department of Innovative Technologies in Medicine and Dentistry, University “G. d’Annunzio”, 66100 Chieti, Italy; valeria.ciummo@graduate.univaq.it

**Keywords:** intestinal fibrosis, fibroblast-to-myofibroblast transition, epithelial-to-mesenchymal transition (EMT), multi-strain probiotics, fibrotic signaling pathway, TGF-β1 signaling, intestinal fibroblasts, intestinal epithelial cells, inflammatory bowel disease (IBD)

## Abstract

Intestinal fibrosis is a common complication of inflammatory bowel diseases (IBD), and, to date, effective and safe antifibrotic drugs are still lacking. Emerging evidence suggests that probiotics may provide novel strategies to counteract fibrotic processes. In this study, we evaluated the anti-fibrotic potential of a multi-strain probiotic formulation, *OxxySlab*^TM^, using in vitro models of intestinal fibrosis and epithelial-to-mesenchymal transition (EMT). Human intestinal fibroblasts (CCD-18Co cell line) and epithelial cells (Caco-2 cell line, IECs) were stimulated with transforming growth factor-β1 (TGF-β1) to induce fibrotic and EMT phenotypes, respectively. Treatment with *OxxySlab* modulated cell proliferation and fibrosis-related markers, which we assessed through CCK-8 assay, Western blotting, and immunofluorescence. The probiotic lysate inhibited both canonical and non-canonical TGF-β1 signaling pathways, and it also reduced TGF-β1 gene expression in activated myofibroblasts, as shown by RT-qPCR. Furthermore, probiotic treatment reversed EMT features by restoring epithelial markers and downregulating mesenchymal markers. These findings highlight the beneficial effects of the multi-strain probiotic formulation as an adjunctive therapeutic agent targeting key pathways involved in intestinal fibrosis.

## 1. Introduction

Intestinal fibrosis is a common pathological consequence of inflammatory bowel diseases (IBD), primarily Crohn’s disease (CD) and ulcerative colitis (UC). In CD, fibrotic strictures and obstructive symptoms affect over 50% of patients during their lifetime, often requiring surgical interventions. In UC, although less frequent, fibrosis can lead to motility disturbances and bowel urgency [1,2]. Despite advances in IBD treatment, effective therapies targeting intestinal fibrosis remain lacking. Fibrosis in IBD is a complex process initially triggered by inflammation but subsequently can progress independently over time. The exact mechanisms of this chronic progression remain unknown.

Current anti-inflammatory treatments, including advanced therapies, do not significantly interrupt the evolution of intestinal fibrosis. The antifibrotic effects of available anti-inflammatory drugs have been evaluated only in limited clinical studies [1,3]. Our previous in vitro investigations revealed that only some of these drugs counteract the transforming growth factor-β1 (TGF-β1)-induced fibrotic responses, making the first direct evaluation of antifibrotic activity in in vitro models of intestinal fibrosis and epithelial-to-mesenchymal transition (EMT) [4].

Intestinal fibrosis is characterized by an abnormal deposition of extracellular-matrix (ECM) proteins produced by activated myofibroblasts at the level of the intestinal tract involved by chronic inflammation. Experimental studies have shown that deletion of myofibroblasts significantly reduces fibrosis, highlighting their role in the disease’s progression [5]. Among fibrotic mediators, TGF-β1 plays a central role by activating intestinal mesenchymal cells and promoting their differentiation into myofibroblasts, which are crucial for ECM buildup [6]. In addition, myofibroblasts can originate from intestinal epithelial cells (IECs) that undergo EMT, a process where the epithelial cells acquire mesenchymal traits, contributing significantly to fibrogenesis [7]. Moreover, the IECs, along with intestinal mucus and the tight junctions—specialized structures capable of holding the cells together [8]—constitute the intestinal mechanical and physical barrier, which is essential for maintaining intestinal homeostasis. The intestinal epithelium plays a crucial role as an interface between the intestinal compartment and the external environment, encompassing bacteria, toxins, and other extraneous compounds. Additionally, it mediates the interaction between luminal factors and the immune system, which is crucial for preserving the mucosal immunity. Then, when any disturbances cause alterations to the epithelium, its barrier function is compromised, leading to intestinal permeability and mucosal inflammation. In this context, impaired integrity causes a disequilibrium between antigens and the local immune system, leading to the translocation of soluble factors, bacterial components, or whole bacteria across the intestinal wall and then promoting the inflammatory process. This phenomenon serves as the basis for the onset and advancement of chronic inflammatory diseases. Of note, the involvement of an impaired intestinal barrier in the pathogenesis of intestinal inflammation has been supported by several studies with animal models [9,10,11,12,13] and has been observed in IBD patients but also in subjects with different gut illnesses [14,15,16].

The gut microbiota is increasingly recognized as a key player in the IBD pathogenesis. Probiotics and their bioactive compounds are being explored as adjunctive therapies to restore intestinal barrier function and modulate inflammation [17,18,19,20,21]. The gut microbiota and its metabolites exert a crucial influence on immune homeostasis, where imbalances of this complex microbial community associated with the human host, called dysbiosis, are frequently associated with the exacerbation of inflammatory processes. Numerous studies have linked disturbances of the gut microbiota to immune-mediated inflammatory diseases, such as Crohn’s disease (CD), ulcerative colitis (UC), multiple sclerosis (MS), and rheumatoid arthritis (RA) [22]. Additionally, the role of the gut microbiota in the pathogenesis of inflammatory diseases, including asthma, type 1 and type 2 diabetes mellitus (DM), and obesity, has been elucidated by several reports [23,24,25]. Dysbiosis is often characterized by a reduction in beneficial bacteria such as *Faecalibacterium* and *Bifidobacterium*. Furthermore, altered levels of key microbial metabolites, including short-chain fatty acids (SCFAs), bile acids (BAs), tryptophan derivatives, and vitamins, play a fundamental role in immune regulation [26]. The microbial metabolism of tryptophan and BAs modulates immune responses via receptors like aryl hydrocarbon receptor (AhR) and Takeda G protein-coupled receptor 5 (TGR5), and disruptions in these pathways are linked to chronic inflammation. Inflammatory conditions and impairments in the protective action exerted by the microbiota often favor the proliferation of pathogenic microbes or the overgrowth of microbial groups such as Enterobacteriaceae, which produce pro-inflammatory factors. This further disrupts the balance of the microbiota, creating a feedback loop that can exacerbate disease states, including IBD, irritable bowel syndrome (IBS), and various metabolic disorders. Research indicates that decreased levels of SCFAs can weaken the integrity of the gut barrier, enabling pathogenic bacteria and their products, such as lipopolysaccharide (LPS), to enter systemic circulation, thereby increasing systemic inflammation [27]. Therapeutic strategies, including the use of probiotics, fecal microbiota transplantation (FMT), and dietary modulation, aimed to restore microbiota balance and microbial metabolite production to mitigate inflammation [22].

However, their role in controlling intestinal fibrosis remains poorly understood, with few studies addressing their effects in fibrosis [28,29]. Our group previously demonstrated that a soluble lysate from the high-concentration multi-strain probiotic Vivomixx^®^ inhibited TGF-β1-induced fibrosis in CCD-18Co [30]. Active compounds of bacterial origin provide significant improvements in safety, stability, and effectiveness compared to live probiotic products, marking a crucial advancement in microbiota-based therapeutics. A primary advantage of bioactive bacterial compounds lies in their enhanced safety profile. While live probiotic preparations are generally safe, they can pose risks, particularly for vulnerable populations. Concerns include the potential for systemic infections, especially in immunocompromised individuals, resulting from bacterial translocation or uncontrolled proliferation. The risk of horizontal gene transfer, potentially disseminating antibiotic resistance genes, and the unpredictable elicitation of adverse immune responses or dysbiosis, also persists with live cultures. In contrast, bioactive compounds are non-viable and often consist of purified molecules or cellular components. This intrinsic property minimizes the aforementioned risks. They eliminate the dangers associated with live bacterial growth, such as infections, and circumvent issues related to gene transfer or uncontrolled proliferation, making them considerably safer for sensitive patient cohorts, including neonates, the elderly, and the immunocompromised [31].

Stability is essential for consistent effectiveness and practical use of probiotic products. Live probiotic products face substantial challenges in maintaining cellular viability and metabolic activity throughout their lifecycle. Susceptibility to environmental stressors—such as oxygen, temperature fluctuations, and the harsh acidic and enzymatic conditions of the gastrointestinal tract—necessitates complex manufacturing, specialized packaging, and stringent storage. This often leads to significant reductions in viable cell counts by the time they reach the target site, resulting in unpredictable dosing and inconsistent therapeutic outcomes [32]. Bioactive bacterial compounds, conversely, demonstrate superior stability. As non-living molecules, they exhibit greater resistance to degradation caused by environmental factors, extreme pH, and enzymatic activity. This enhanced resilience translates into extended shelf life, simplified storage and transportation (often without refrigeration), and, crucially, a more consistent and precise delivery of the active compound to the host, ensuring reliable and reproducible clinical effects [33].

Furthermore, bioactive bacterial compounds provide a more precise and potentially more effective method for achieving beneficial outcomes [34]. The beneficial effects attributed to live probiotics are frequently mediated not by the live bacteria themselves, but by their secreted metabolites or structural components—precisely what postbiotics represent. These compounds include vital molecules such as SCFAs, bacteriocins, exopolysaccharides, and various vitamins. By directly administering these characterized compounds, the need for bacterial colonization and viability can be avoided, allowing the delivery of targeted therapeutic agents more effectively [35]. The direct delivery of bioactive compounds provides for a more targeted mechanism of action. These compounds can be carefully chosen based on their known biological activities, such as butyrate’s role in gut barrier integrity or bacteriocins’ antimicrobial properties. The defined chemical nature of these bioactive compounds also facilitates rigorous standardization and quality control, which leads to greater predictability in therapeutic outcomes compared to the strain-specific and batch-to-batch variations inherent in live probiotic products. This precision and consistency are fundamental for their integration into evidence-based medicine, paving the way for more controlled, safer, and highly effective microbiota-based interventions [31].

Recently, we investigated another formulation, *OxxySlab*^TM^ (formerly SLAB51), which attenuated LPS-induced inflammation in Caco-2 IECs [36]. *OxxySlab* is a multispecies probiotic formulation that contains eight lyophilized bacterial strains. It has demonstrated therapeutic potential in various pathological conditions [37,38,39,40], including Alzheimer’s disease [41,42,43], Parkinson’s disease [44,45], COVID-19 [46,47], sleep deprivation [48], neonatal hypoxia [49], and acclimatization to high altitude [50], primarily through microbiota modulation and anti-inflammatory effects.

The present study aimed to evaluate the antifibrotic effects of *OxxySlab* in in vitro models of intestinal fibrosis, focusing on the modulation of fibrotic and EMT markers and the regulation of TGF-β1 signaling pathways.

## 2. Materials and Methods

### 2.1. Cell Lines

Human intestinal CCD-18Co fibroblast cell line (CRL-1459) was achieved from American Type Culture Collection (ATCC, Georgetown, DC, USA) and then cultured in Dulbecco’s modified Eagle’s medium (DMEM) that was supplemented with 10% of Fetal Bovine Serum (FBS), 100 U/mL penicillin, 100 mg/mL streptomycin, and 2 mM glutamine. After reaching 80% confluence, the cells were detached with trypsin solution from bovine pancreas (Euro Clone, West York, UK), sub-cultured and seeded at 7000 cells/cm^2^ in a sterile tissue culture flask for the following experiments.

The Caco-2 cell line acquired from Sigma-Aldrich (St. Louis, MO, USA) was cultured in flasks, in DMEM, supplemented with 10% of FBS, 2 mM L-glutamine, 100 U/mL penicillin, 100 μg/mL streptomycin, 1% non-essential amino acids, and refreshed every two days. After reaching 80% confluence, cells were detached with trypsin and seeded at 60,000 cells/cm^2^ into sterile tissue culture 12-well plates (Becton, San Jose, CA, USA) for differentiation and experimental conditions.

Both cell lines were cultured in a humidified atmosphere of 95% air and 5% CO_2_ at 37 °C. All the reagents for cell biology and consumables were acquired from EuroClone (EuroClone, West York, UK) if not otherwise described.

### 2.2. Preparation of Bacterial Lysate

The *OxxySlab™* multi-strain probiotic formulation (EOS2021 Srl, Ardea, Rome, Italy) developed by Professor Claudio De Simone, includes eight lyophilized bacterial strains of lactic acid bacteria and bifidobacteria as follows: Streptococcus *thermophilus* CNCM I-5570, *Lactobacillus brevis* CNCM I-5566, *Bifidobacterium animalis subsp. lactis* CNCM I-5571, *Bifidobacterium animalis subsp. lactis* CNCM I-5572, *Lactobacillus plantarum* CNCM I-5569, *Lactobacillus paracasei* CNCM I-5568, *Lactobacillus acidophilus* CNCM I-5567, *Lactobacillus helveticus* CNCM I-5573.

The bacterial lysate was obtained as described in our previous works [30,36]. Briefly, 133 × 10^9^ CFU of lyophilized probiotic formulation were resuspended in 10 mL of cool phosphate-buffered saline (PBS, EuroClone, West York, UK), centrifuged at 8600× *g*, and washed twice. The bacterial resuspension was sonicated with a Vibracell sonicator (Sonic and Materials, Danbury, CT, USA) for 30 min, alternating 10 s of sonication and 10 s of pause. Measuring the absorbance of the sample at 590 nm with a spectrophotometer (Eppendorf, Hamburg, Germany), before and after every sonication step, was performed to ascertain the breakdown of the bacterial cell. Then, the samples were centrifuged at 17,949× *g*, and the supernatants were filtered, in sterility, using a filter with 0.22 µm-pore (Corning Incorporated, Somerville, MA, USA) to eliminate any remaining bacteria. Total protein content was quantified by a *DC* protein assay (Bio-Rad Laboratories, Hercules, CA, USA), using bovine serum albumin (BSA, Sigma Aldrich, St. Louis, MO, USA) in PBS for the standard curve.

### 2.3. In Vitro Cell Models and Treatments

CCD-18Co cells are used to establish an in vitro model of intestinal fibrosis. CCD-18Co were plated at 5000 cells/cm^2^, and the following day, the medium was replaced with serum-deprived medium for 24 h and then stimulated with 10 ng/mL of TGF-β1 (hTGF-β1; Cell Signaling Technology, Danvers, MA, USA) for 48 h to induce the fibrotic phenotype. The cells were treated with TGF-β1 (10 ng/mL) for 48 h in the presence or absence of probiotic formulation lysate at different concentrations. Non-treated starved cells were referred to as “Control” in all experiments.

To establish an EMT in vitro model, the Caco-2 cell line was seeded at 60,000 cells/cm^2^ and cultured in sterile tissue culture 12-well plates to allow the cells to spontaneously differentiate, express morphological (polarized columnar epithelium) and functional characteristics of the intestinal epithelial cells (IECs) [51]. After fourteen days post-confluence, Caco-2 IECs were treated with TGF-β1 (20 ng/mL) in the serum-free medium up to 96 h with or without the multi-strain probiotic lysate at different concentrations. Then, the cells were harvested and centrifuged at 400× *g* for 10 min. The cells were counted by an inverted research microscope (Eclipse 50i, Nikon, Tokyo, Japan).

### 2.4. Cell Proliferation Assay of CCD-18Co

The cell proliferation of CCD-18Co cells was measured using the Cell Counting Kit-8 (CCK-8) (Sigma-Aldrich) according to the manufacturer’s instructions. The 96-well-plated CCD-18Co cells were treated with different concentrations of the probiotic lysate: *OxxySlab* (25–50–100 µg protein/mL) with or without stimulation by the TGF-β1. After treatment, 10 µL of CCK-8 assay reagent was added to each well at time points of 0, 24, and 48 h. The optical density (OD) values were measured using a spectrophotometer (BioRad, Hercules, CA, USA) at 450 nm.

### 2.5. Cell Viability Assay of Caco-2 IECs

Cell viability for the Caco-2 IECs was assessed with the 3-(-2,5 diphenyl tetrazolium bromide (MTT) assay (Immunological Sciences, Rome, Italy). Caco-2 cell line was seeded into sterile tissue culture 96-well plates at 60,000 cells/cm^2^, to allow the differentiation of the cells as described before. Fourteen days post-confluence, the 96-well-plated Caco-2 IECs were incubated with different concentrations of probiotic lysate (10–25–50–100–200 µg protein/mL). After incubation, the MTT assay was performed at different times, 0, 24, 48, 72, and 96 h, and the absorbance signals were measured using a spectrophotometer at 570 nm and the background at 630 nm. The background absorbances have been subtracted from the signal absorbance to obtain normalized absorbance values.

### 2.6. Western Blot Analysis

For Western blot analyses, CCD-18Co were plated at 5000 cells/cm^2^ in a 25 cm^2^ sterile tissue culture flask, and Caco-2 cells were seeded in 12-well plates at 60,000 cells/cm^2^. After the treatments described before in both in vitro models, cells were washed in PBS and lysed using a cell scraper in RIPA buffer (Merck KGaA, Darmstadt, Germany) containing a protease inhibitor mixture (Merck KGaA, Darmstadt, Germany). The protein content of samples was then quantified by DC Protein Assay using BSA in RIPA buffer as a standard. Subsequently, the protein samples (25 μg), prepared using the sample buffer, were boiled (5 min at 100 °C) and then subjected to electrophoresis using a 10% SDS-polyacrylamide gel. The separated proteins were transferred onto a 0.45 μm nitrocellulose membrane sheet (BioRad) for 1 h at 4 °C at 70 V, using a Mini Trans-Blot Cell apparatus (BioRad). Blocking of membranes was performed by soaking the membrane in 5% non-fat dry milk or 5% BSA for 2 h at room temperature. Overnight incubation was performed at 4 °C with several antibodies reported in Table 1. Horseradish peroxidase (HRP)-conjugated goat anti-rabbit and HRP-conjugated rabbit anti-mouse were used as secondary antibodies (Millipore EMD, Darmstadt, Germany). Immuno-reactive protein bands were visualized by enhanced chemiluminescence (ECL, Amersham Pharmacia Biotech, Amersham, UK) according to the manufacturer’s instructions. Densitometry analyses were determined using the ALLIANCE (UVITEC, Cambridge, UK) chemiluminescence documentation system and normalized to the relative GAPDH or β-Tubulin bands.

### 2.7. Immunofluorescent Staining

CCD-18Co and Caco-2 IECs, grown on coverslips in a 12-well plate (seeded at 5000 cells/cm^2^ and 60,000 cells/cm^2^, respectively), were stimulated as previously reported. After the respective treatments, the coverslips were then washed with PBS, fixed with 4% formaldehyde for 20 min, and permeabilized in 0.1% Triton X-100 (Sigma-Aldrich) for 5 min, except for the evaluation of the occludin and E-cadherin, which did not require permeabilization. Subsequently, the coverslips were blocked with 3% BSA in PBS for 20 min at room temperature. Then, cells were maintained overnight at 4 °C with specific primary antibodies listed in Table 2. Therefore, staining was performed using FITC-conjugated goat anti-mouse secondary antibody (Bethyl Laboratories, Inc, Montgomery, AL, USA) or FITC-conjugated goat anti-rabbit secondary antibody (Millipore) at a 1:1000 dilution for 1 h at room temperature. CCD-18Co were also incubated with TRITC labeled phalloidin (Sigma-Aldrich) for 45 min at room temperature. Then, the coverslips were mounted with VECTASHIELD^®^ Antifade Mounting Medium with DAPI (Vector Laboratories, Inc., Burlingame, CA, USA) and examined at 40× magnification with a fluorescent microscope (Eclipse Ts2R-FL, Nikon Corporation, Tokyo, Japan).

### 2.8. Total RNA Extraction and Quantitative Real-Time PCR (qPCR)

Real-time RT-PCR using a ViiA7 sequence detection system (Applied Biosystems, Foster City, CA, USA) was carried out to evaluate the TGF-β1 gene expression in untreated (control) and treated CCD-18Co. Total RNA was extracted from the cells with the RNeasy Mini Kit (QIAGEN, Hilden, Germany) following the manufacturer’s instructions and quantified by spectrophotometry. 1 μg of total RNA was reverse transcribed in a final volume of 20 µL using a mixture of random primers as reported below. An amount of 0.5 µg of each cDNA was used to perform real-time PCR. Real-time quantitative RT-PCR analysis was carried out by SYBR Green dye detection (Thermo Fisher Scientific, Waltham, MA, USA). Reverse and forward primers, acquired from IDT (acquired from Integrated DNA Technologies, IDT, Coralville, IA, USA), were used at a concentration of 1 µM (hTGF-β1), and their sequences were as follows: hTGF-β1 forward 50-CAACGAAATCTATGACAAGTTCAAGCAG-30 and reverse 50-CTTCTCGGAGCTCTGATGTG-30. The mRNA level of GAPDH was used as an internal control for the normalization with subsequent primers: GAPDH forward 50-TTGCCCTCAACGACCACTTT-30 and reverse 50-TGGTCCAGGGGTCTTACTCC-30. The fold-change quantification in target genes was calculated with the 2^− DDCt^ method. The samples were run in triplicate and repeated twice.

### 2.9. Statistical Analysis

All data were statistically evaluated by GraphPad Prism (version 8.02, GraphPad Software, San Diego, CA, USA). One-way ANOVA or two-way ANOVA and Dunnett post hoc test were used. Data were expressed as mean ± SD or mean ± SEM. The *p*-values were considered statistically significant when lower than 0.05.

## 3. Results

### 3.1. Multi-Strain Probiotic Lysate Reduced CCD-18Co Cell Proliferation

**Figure 1:**
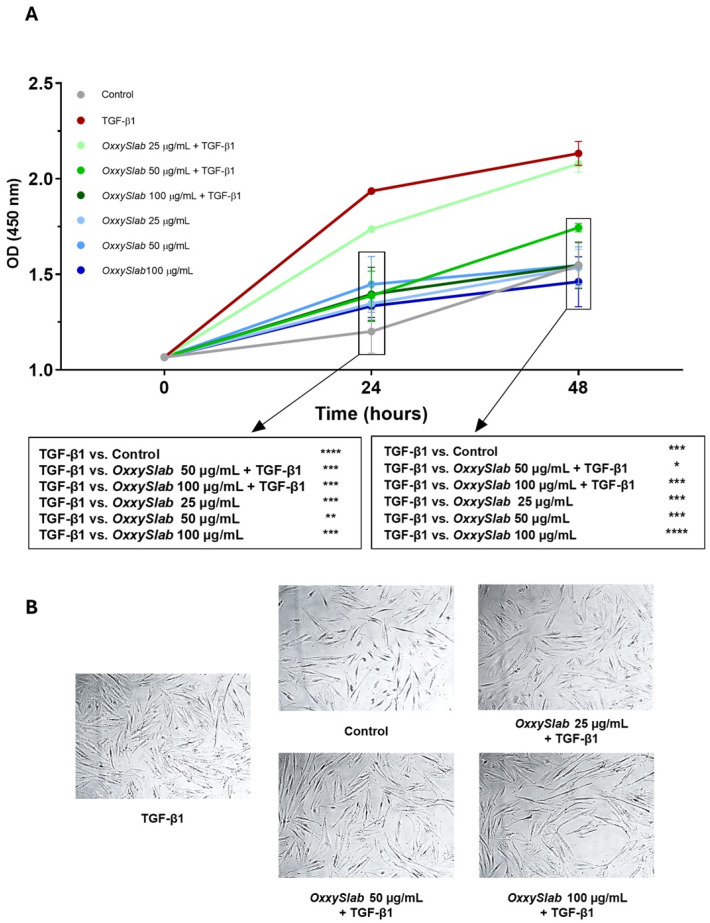
Effect of *OxxySlab* lysate on TGF-β1-induced proliferation of CCD-18Co. (**A**) CCK-8 assay on CCD-18Co cells stimulated with TGF-β1 alone or in combination with *OxxySlab* lysate (25, 50, and 100 μg protein/mL) for 48 h, after 24 h of starvation. Data from two independent experiments performed in triplicate are expressed as mean ± SEM and analyzed by two-way ANOVA. All data were compared with the TGF-β1 condition (* *p* < 0.05, ** *p* < 0.01, *** *p* < 0.0001, **** *p* < 0.0001). (**B**) Phase-contrast images of CCD-18Co cells after 48 h of treatment: Control, TGF-β1 alone, and TGF-β1 in combination with 25, 50, and 100 μg/mL of the probiotic lysate, respectively.

To assess the anti-fibrotic potential of the multi-strain probiotic formulation *OxxySlab*, we evaluated the proliferation of CCD-18Co cells following stimulation with TGF-β1 at 10 ng/mL for 48 h, in the presence or absence of increasing concentrations (25–50–100 μg protein/mL) of probiotic lysate. Cell proliferation was measured using the CCK8 assay (Figure 1A). As expected, TGF-β1 stimulation significantly increased fibroblast proliferation compared to untreated controls. Co-treatment with probiotic lysate at 50 and 100 µg/mL attenuated the proliferative response, restoring cell proliferation levels comparable to the control group. Notably, treatment with probiotic lysate alone did not significantly alter basal proliferation rates. These findings were corroborated by phase-contrast microscopy, which revealed morphological changes consistent with reduced cell density in the probiotic-treated groups (Figure 1B). Based on its lack of significant effect, the 25 μg/mL concentration was excluded from subsequent experiments.

### 3.2. Multi-Strain Probiotic Lysate Downregulated Fibrotic Marker Expression on TGF-β1-Stimulated CCD-18Co Cells

**Figure 2:**
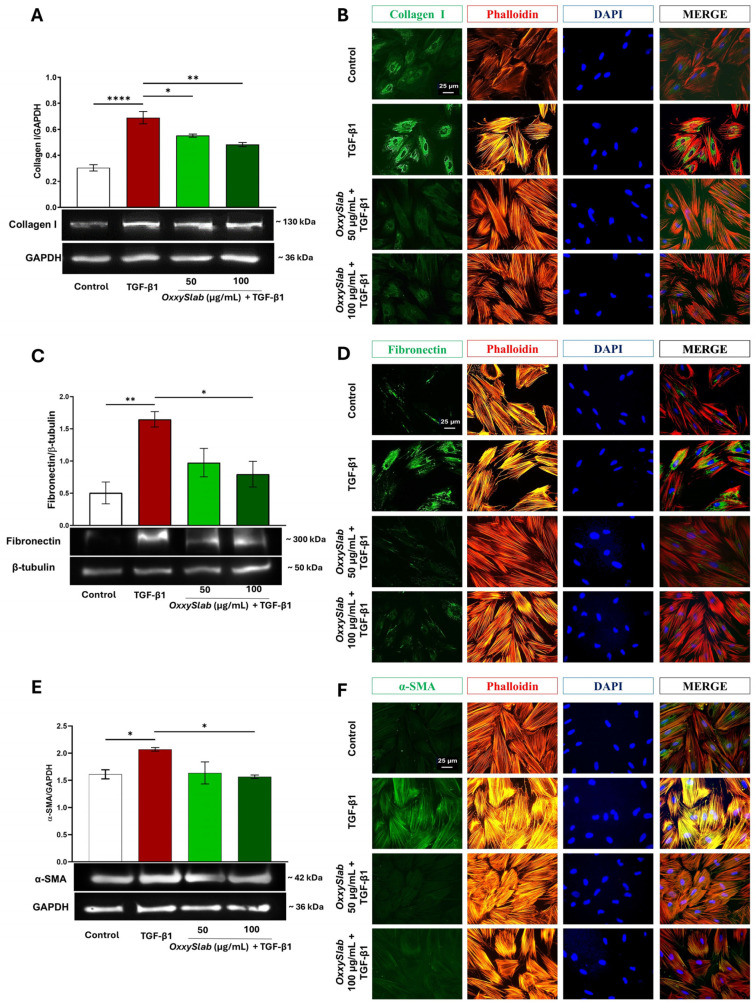
Effect of *OxxySlab* on fibrosis markers in in vitro intestinal fibrosis model. Immunoblotting assay for (**A**) collagen I, (**C**) fibronectin, and (**E**) α-SMA performed on CCD-18Co incubated for 48 h with TGF-β1 (10 ng/mL) in the presence or absence of probiotic lysate (50–100 µg protein/mL). Densitometric analysis was normalized to GAPDH for collagen I and α-SMA, and to β-tubulin for fibronectin. Data are from three independent experiments and are expressed as mean ± SEM. For comparative analysis of data, a one-way ANOVA is used. (* *p* < 0.05, ** *p* < 0.01, **** *p* < 0.0001). Representative immunofluorescence images of untreated and treated CCD-18Co, as above described, stained with antibodies against (**B**) collagen I, (**D**) fibronectin, and (**F**) α-SMA (green), and TRITC-phalloidin to F-actin (red). Nuclei were counterstained with DAPI (blue) (magnification 40×).

Fibroblast activation status is a key event in intestinal fibrogenesis [30,52] characterized by cytoskeleton remodeling and upregulation of fibrotic markers, including collagen type I, fibronectin, and alpha-smooth muscle actin (α-SMA) protein. To evaluate the impact of probiotic lysate treatment, CCD-18Co cells were stimulated with TGF-β1 and analyzed for fibrotic marker expression by Western blot. As expected, TGF-β1 stimulation induced a marked increase in collagen I and fibronectin protein levels compared to untreated controls. Co-treatment with the multi-strain probiotic lysate significantly attenuated this upregulation in a concentration-dependent manner (Figure 2A,C), indicating a suppressive effect on fibroblast activation. These findings suggest that OxxySlab modulates key components of the fibrotic response, potentially interfering with the signaling pathways driving ECM protein overproduction.

We further evaluated the expression of α-SMA in TGF-β1-activated CCD-18Co exposed to probiotic lysate. Western blot analysis confirmed a significant upregulation of α-SMA following TGF-β1 stimulation. Notably, co-treatment with the probiotic lysate resulted in a concentration-dependent reduction in α-SMA levels, with the highest concentration showing a statistically significant effect (Figure 2E). Immunofluorescence staining corroborated these findings, revealing increased signal intensity for collagen I, fibronectin, and α-SMA in TGF-β1-treated cells compared to controls (Figure 2B,D,F). Probiotic co-treatment markedly reduced the staining intensity of these fibrotic markers, restoring levels comparable to untreated cells.

In addition, TGF-β1 stimulation induced pronounced cytoskeleton remodeling, as evidenced by enhanced phalloidin staining and the formation of prominent stress fibers across the cytoplasm. These features were absent in control cells. Co-treatment with probiotic lysate attenuated this effect, reducing phalloidin fluorescence and restoring cytoskeletal organization to near-baseline levels.

Overall, these results demonstrate that the multi-strain probiotic lysate exerts remarkable anti-fibrotic effects by simultaneously inhibiting fibroblast proliferation and expression of collagen and fibronectin, as well as reversing cytoskeletal alterations associated with fibrogenesis.

### 3.3. Probiotic Lysate Interferes with Activation of TGF-β1 Signaling Pathways

**Figure 3:**
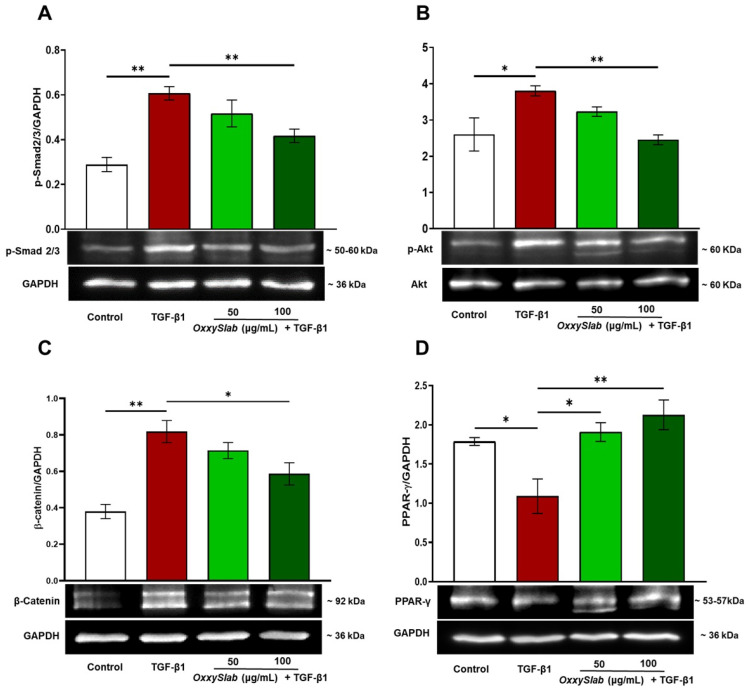
*OxxySlab* effect on canonical and non-canonical TGF-β pathway in in vitro intestinal model. Analysis and representative images of immunoblotting of (**A**) p-Smad2/3, (**B**) pAkt, (**C**) β-Catenin, (**D**) PPAR-γ in CCD-18Co starved for 24 h, then incubated for 48 h with TGF-β1 (10 ng/mL) in the presence or absence of two selected concentrations of the probiotic lysate (50–100 μg protein/mL). Densitometric analysis was performed by normalizing vs. GAPDH, except for p-Akt, which was normalized to the respective Akt. Data are from three independent experiments and are expressed as mean ± SEM. For the analysis of data, one-way analysis of variance ANOVA (* *p* < 0.05, ** *p* < 0.01).

The activation of intestinal fibroblasts, a key player of fibrosis, is triggered by chronic inflammation and is regulated by multiple cytokines, with TGF-β1 playing a central role. TGF-β1 exerts its effects through both Smad-dependent (canonical) and Smad-independent (non-canonical) signaling pathways. To investigate whether the antifibrotic effects of the probiotic formulation lysate were mediated by modulation of the canonical Smad pathway, we performed Western blot analysis to assess the levels of phosphorylated Smad 2/3 (p-Smad 2/3). As shown in Figure 3A, TGF-β1 stimulation markedly increased Smad2/3 phosphorylation compared to untreated cells, in parallel with the upregulation of fibrotic markers. Importantly, co-treatment with the probiotic lysate at both tested concentrations significantly reduced p-Smad2/3 levels, suggesting an attenuation of TGF-β1-induced transcriptional activation of profibrotic genes. This reduction was consistent with the observed downregulation of collagen I, fibronectin, and α-SMA protein expression.

In addition to the canonical Smad pathway, TGF-β1 also activates the non-canonical PI3K/Akt pathway [53,54,55]. To evaluate the involvement of this pathway, we analyzed the phosphorylation status of Akt by Western blot. As shown in Figure 3B, TGF-β1 stimulation significantly increased phosphorylated-Akt (p-Akt) levels compared to control cells. Sustained Akt activation is known to promote excessive ECM production and drive fibroblast-to-myofibroblast differentiation, thereby exacerbating intestinal fibrosis [56]. Remarkably, co-treatment with the probiotic lysate markedly reduced p-Akt expression, indicating a modulatory effect on this signaling axis.

We further investigated two additional pathways implicated in TGF-β1-mediated fibrogenesis: the WNT/β-catenin pathway, which promotes fibrosis, and the PPAR-γ pathway, which exerts anti-fibrotic effects [57]. TGF-β1 stimulation led to a significant increase in β-catenin protein levels (Figure 3C), consistent with its pro-fibrotic role. This upregulation was effectively suppressed by probiotic lysate treatment. Conversely, TGF-β1 reduced PPAR-γ expression, while co-treatment with the probiotic lysate restored its levels (Figure 3D), suggesting a protective regulatory effect. Taken together, these findings demonstrated that the multi-strain probiotic formulation attenuates intestinal fibrosis by interfering with multiple intracellular signal transduction pathways activated by TGF-β1, including Smad-dependent signaling (p-Smad 2/3) and Smad-independent pathways (PI3K/Akt, WNT/β-catenin, and PPAR-γ).

### 3.4. OxxySlab Modulates TGF-β1 Gene Expression

**Figure 4:**
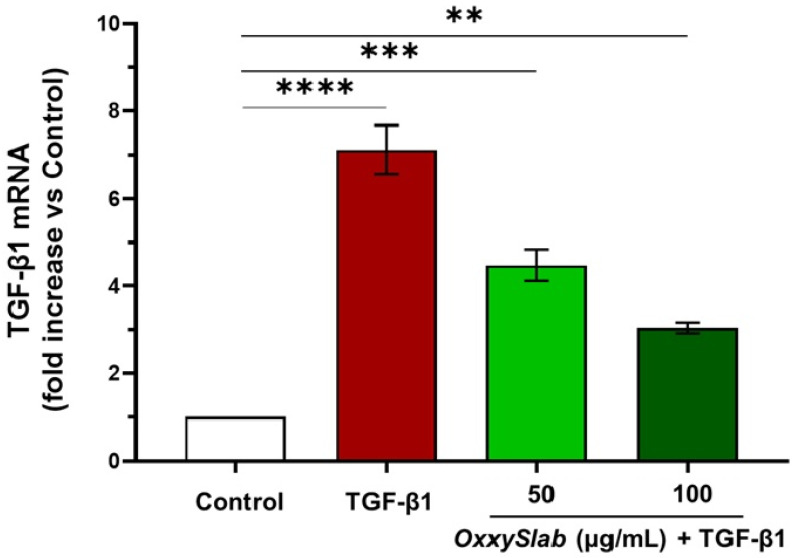
OxxySlab effect on TGF-β1 gene expression in intestinal fibrosis model. The SYBRGreen Real-Time PCR analysis of the TGF-β1 gene was performed on CCD-18Co. The mRNA levels were relative to the amount of GAPDH mRNA. Data from three independent experiments in triplicate are shown as mean  ±  SEM. The data shown were obtained from one-way ANOVA followed by Dunnett’s multiple comparisons test (** *p* < 0.01, *** *p* < 0.001, **** *p* < 0.0001 vs. Control).

To evaluate whether the probiotic formulation influences transcriptional regulation, we analyzed TGF-β1 gene expression in CCD-18Co cells by RT-qPCR. Consistent with previous findings [58], TGF-β1 stimulation significantly upregulated its own gene expression, reflecting a positive feedback loop that sustains fibrogenesis. Treatment with *OxxySlab* lysate markedly reduced TGF-β1 mRNA levels in a concentration-dependent manner (Figure 4), suggesting that the probiotic formulation lysate interferes with the autocrine amplification of TGF-β1 signaling. This transcriptional downregulation further supports the observed attenuation of fibrotic marker expression and signaling pathway activation.

### 3.5. Effect of the Multi-Strain Probiotic Lysate on Caco-2-IECs Viability

**Figure 5:**
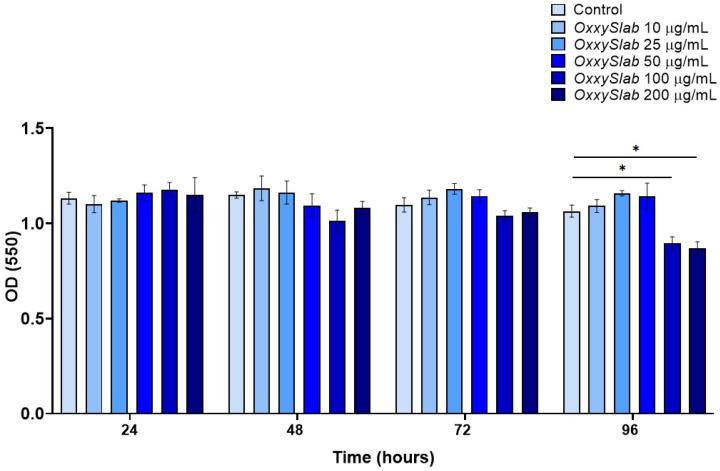
Cell viability assay (MTT) in Caco-2 IECs incubated with *OxxySlab* lysate (10–25–50–100–200 μg protein/mL) for 96 h. Data from three independent experiments performed in triplicate are expressed as mean ± SEM and then analyzed with the two-way ANOVA (* *p* < 0.05 vs. Control).

Prior to evaluating the impact of the probiotic formulation lysate on epithelial-to-mesenchymal transition (EMT), we assessed the viability of Caco-2 intestinal epithelial cells (IECs) following exposure to increasing concentrations of *OxxySlab* lysate (25, 50, 100, and 200 μg/mL) for up to 96 h. Cell viability was measured using the MTT assay. As shown in Figure 5, a significant reduction in absorbance, indicative of decreased cell number, was observed at the two highest concentrations (100 and 200 μg/mL), but this reduction was only observed at the 96 h time point. No cytotoxic effects were detected at earlier time points or at lower concentrations. Based on these findings, the 100 and 200 μg/mL concentrations of *OxxySlab* were excluded from subsequent EMT experiments to avoid confounding effects related to reduced cell viability.

### 3.6. OxxySlab Attenuates TGB-β1-Induced EMT in Caco-2 IECs

**Figure 6:**
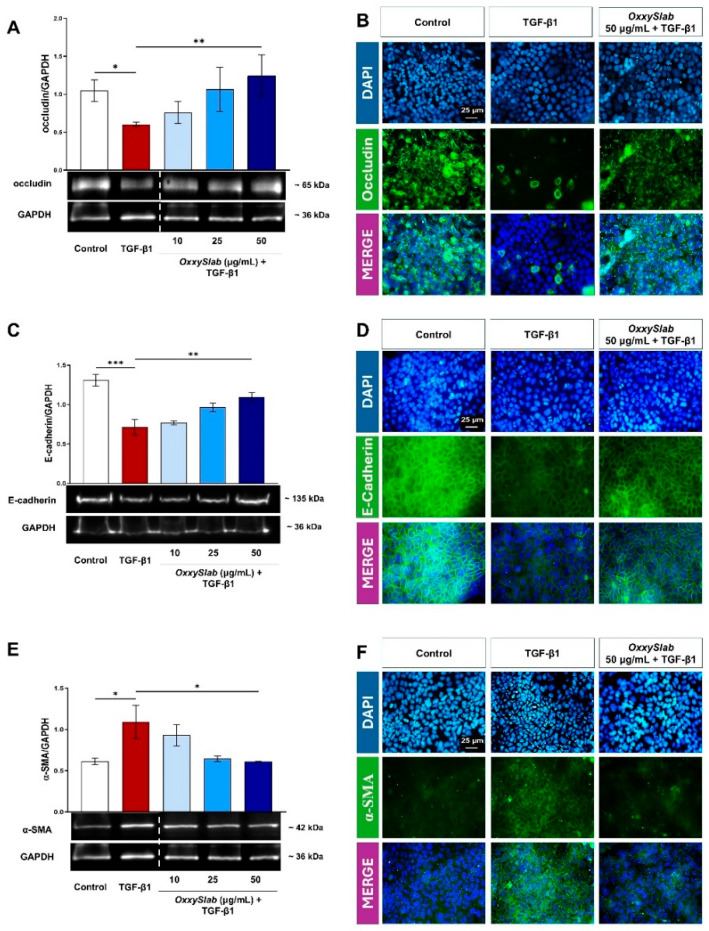
Effect of *OxxySlab* on epithelial and mesenchymal markers expression in the in vitro EMT model. Immunoblotting assay for (**A**) occludin and (**C**) E-cadherin, and (**E**) α-SMA was performed on Caco-2 IECs incubated for 96 h with TGF-β1 (20 ng/mL) alone or in combination with *OxxySlab* lysate at selected concentrations (10–25–50 µg protein/mL). Representative images of immunoblotting for occludin, E-cadherin, α-SMA, and GAPDH are shown. The dotted lines indicate areas in which the images were cropped to remove lanes irrelevant for this study. Densitometric analysis was normalized to GAPDH. Data are from three independent experiments, and values are expressed as mean ± SEM. Statistical analysis was performed using one-way ANOVA (* *p* < 0.05, ** *p* < 0.01, *** *p* < 0.001). No significant difference was detected between TGF and its combination with the lowest doses of probiotics. Representative immunofluorescence images from three independent experiments in duplicate, of untreated and treated Caco-2 IECs stained with antibodies against (**B**) occludin, (**D**) E-cadherin, (**F**) α-SMA antibodies (green), are shown. Nuclei were counterstained with DAPI (blue) (magnification 40×).

EMT is a key contributor to the expansion of the intestinal myofibroblast population, driven by the transdifferentiation of epithelial cells into mesenchymal-like cells. TGF-β1 is recognized as the most potent inducer of EMT in intestinal epithelial cells [59]. To evaluate the effect of the probiotic formulation lysate on EMT, we analyzed the expression of two epithelial markers: occludin, a tight junction protein critical for barrier integrity, and E-cadherin, a key component of the apical junctional complex responsible for cell–cell adhesion. Caco-2 IECs were incubated with TGF-β1 for 96 h in the presence or absence of *OxxySlab* lysate at selected concentrations (10, 25, and 50 μg/mL). Western blot analysis revealed that TGF-β1 stimulation significantly reduced occludin (Figure 6A) and E-cadherin protein levels (Figure 6C), consistent with EMT induction. Co-treatment with the probiotic lysate restored the expression of both markers in a concentration-dependent manner. These findings were further confirmed by immunofluorescence staining, which showed reduced signal intensity for occludin (Figure 6B) and E-cadherin (Figure 6D) in TGF-β1-treated cells, and a marked recovery upon probiotic treatment. These results suggest that *OxxySlab* preserves epithelial identity and barrier function by counteracting TGF-β1-induced EMT.

In addition to the downregulation of epithelial markers, TGF-β1 stimulation of Caco-2 IECs resulted in a significant upregulation of the mesenchymal cell marker α-SMA, consistent with EMT induction. Treatment with the probiotic lysate resulted in a dose-dependent reduction in α-SMA expression, with the highest concentration showing a statistically significant effect (Figure 6E). These findings were further supported by immunofluorescence analysis, which confirmed a decrease in α-SMA signal intensity in cells treated with the probiotic lysate (Figure 6F).

**Figure 7:**
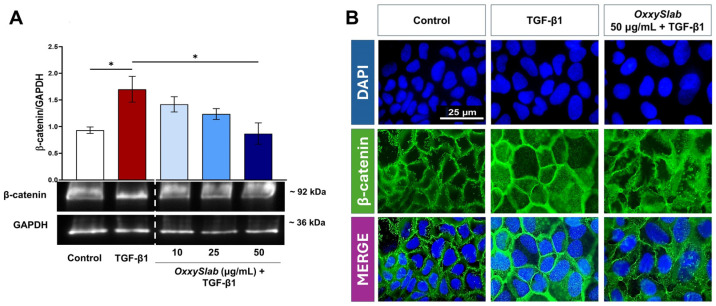
Effect of *OxxySlab* on β-catenin expression in the in vitro EMT model. Immunoblotting assay for (**A**) β-catenin was performed on Caco-2 IECs incubated for 96 h with TGF-β1 (20 ng/mL) alone or in combination with *OxxySlab* lysate at selected concentrations (10–25–50 µg protein/mL). Representative images of immunoblotting for β-catenin and GAPDH are shown. The dotted lines indicate areas in which the images were cropped to remove lanes irrelevant for this study. Densitometric analysis was normalized to GAPDH. Data are from three independent experiments, and values are expressed as mean ± SEM. Statistical analysis was performed using one-way ANOVA (* *p* < 0.05). No significant difference was detected between TGF-β1 and its combination with the lowest doses of probiotics. Representative immunofluorescence images of untreated and treated Caco-2 IECs stained with antibodies against (**B**) β-catenin antibodies (green), are shown. Nuclei were counterstained with DAPI (blue) (magnification 100×).

To further elucidate the mechanism by which *OxxySlab* lysate inhibits EMT, we investigated the involvement of the WNT/β-catenin signaling pathway. Canonical WNT signaling promotes EMT by increasing β-catenin levels, thereby enhancing the EMT-related transcription [59]. Western blot and densitometric analysis revealed that TGF-β1 stimulation significantly elevated β-catenin expression in Caco-2 cells (Figure 7A). Additionally, we also observed a higher nuclear localization of this protein in TGF-β1-stimulated cells as detected by immunofluorescence (Figure 7B). Co-treatment with the probiotic lysate effectively counteracted this increase, as also evidenced by a reduced signal in the nuclear compartment. These findings suggest that OxxySlab modulates WNT/β-catenin signaling to suppress the EMT process.

Taken together, these results demonstrate that the multi-strain probiotic formulation mitigates intestinal fibrosis by reducing the EMT process, restoring epithelial marker expression, and reducing mesenchymal marker levels.

## 4. Discussion

Intestinal fibrosis is a severe complication of IBD, including Crohn’s disease and UC, triggered by chronic inflammation. This pathological process is characterized by an abnormal ECM deposition leading to the formation of intestinal strictures and obstruction. Despite its significant clinical impact, no safe and effective antifibrotic agents have been approved to date, and surgical intervention remains the only option in advanced cases. The limited understanding of the molecular mechanisms underlying fibrosis initiation and progression continues to hinder the development of effective therapies.

However, recent developments in antifibrotic therapy have broadened the therapeutic landscape, introducing novel strategies that complement traditional approaches. Among these, WNT pathway inhibitors have emerged as promising candidates due to their ability to disrupt fibroblast activation and extracellular matrix deposition—hallmarks of fibrogenesis across organ systems [60,61]. In parallel, PPAR-γ modulation has demonstrated antifibrotic efficacy by attenuating pro-inflammatory and profibrotic signaling, particularly through downregulation of TGF-β and PDGF pathways [62,63]. Additionally, microbiota-targeted interventions, including probiotics and fecal microbiota transplantation, are gaining traction for their capacity to modulate immune responses and oxidative stress, thereby indirectly influencing fibrotic progression [44,61]. Notably, the *SLAB51* formulation has shown systemic anti-inflammatory effects that may extend to fibrotic contexts [44]. These emerging strategies underscore the need for a multidimensional therapeutic framework, within which our findings may be contextualized to inform future translational applications.

Recent evidence has highlighted the therapeutic potential of probiotics in IBD, owing to their ability to modulate inflammation, regulate immune responses, and restore gut microbiota balance [64]. Furthermore, the emerging role of microbiota in IBD-associated fibrosis suggests that microbiota-targeted interventions may offer promising antifibrotic strategies [65]. However, few studies have directly investigated the impact of probiotic strains or their metabolites on fibrotic signaling pathways [66]. In a previous study, we demonstrated that Vivomixx^®^ lysate could antagonize TGF-β1-induced fibrosis in intestinal fibroblasts. The present study aimed to evaluate the antifibrotic potential of *OxxySlab*, a multi-strain probiotic formulation previously shown to exert beneficial effects in various pathological disorders [41,42,43,44,45,46,47,48,49,50].

We demonstrated that *OxxySlab* effectively attenuated the profibrotic fibroblasts’ proliferation, differentiation, activation, and EMT in our in vitro models. In human intestinal fibroblasts (CCD-18Co), TGF-β1 stimulation induced cell proliferation and upregulated fibrotic markers, including collagen I, fibronectin, and α-SMA, along with cytoskeletal reorganization. Co-treatment with probiotic lysate reversed these effects in a dose-dependent manner, suggesting a potent antifibrotic action. Fibrosis is sustained by activated myofibroblasts, which arise from multiple sources and are primarily driven by TGF-β1 signaling. This cytokine activates both Smad-dependent (canonical) and Smad-independent (non-canonical) pathways. Our data showed that probiotic lysate treatment significantly reduced Smad2/3 phosphorylation and downregulated TGF-β1 gene expression, indicating interference with the canonical pathway.

We also explored the PI3K/Akt axis, a TGF-β1 non-canonical pathway implicated in various fibrotic diseases [53,54,55,67]. Stimulation with TGF-β1 increased the levels of p-Akt, while co-treatment with probiotic lysate suppressed this activation, suggesting that *OxxySlab* mitigates fibrosis by modulating Akt signaling.

Further analysis revealed that TGF-β1-induced fibrosis involves crosstalk with the WNT/β-catenin pathway, which promotes fibroblast activation and EMT [58,68,69]. In this study, probiotic lysate decreased β-catenin expression and increased PPAR-γ levels, a nuclear receptor known for its antifibrotic properties [57,68,69,70,71,72,73]. These findings support the hypothesis that *OxxySlab* exerts its effects by antagonizing multiple pro-fibrotic signaling pathways.

Beyond fibroblasts, epithelial cells contribute to fibrosis through EMT, a process characterized by the loss of epithelial markers (E-cadherin, occludin) and the gain of mesenchymal traits (α-SMA, fibronectin) [74,75]. TGF-β1 stimulation of Caco-2 IECs induced EMT, as evidenced by reduced epithelial marker expression and increased α-SMA and β-catenin levels. Interestingly, probiotic treatment was found to reverse these changes, restoring epithelial phenotype and suppressing mesenchymal transition.

We acknowledge that this study has several limitations, including the use of a single fibrotic stimulus and the analysis restricted to the principal markers involved in the EMT. Future research should address these limitations. Specifically, it would be advisable to investigate additional cytokines and environmental factors involved in the development of intestinal fibrosis, as this would enhance the translational relevance of our results. Furthermore, exploring more EMT markers, such as N-cadherin, vimentin, and transcription factors like Snail/Slug and ZEB1/2, would provide a more comprehensive understanding of the effects of the probiotic formulation.

Recent studies on probiotics have increasingly emphasized the differences between viable probiotics, non-viable probiotics, and probiotic compounds, particularly in the realm of microbial biotherapy. Viable probiotics are recognized for their capacity to colonize the gut and provide beneficial effects, such as immune modulation and gut health enhancement. Conversely, non-viable probiotics are gaining attention for their advantages in terms of safety, stability, and efficacy, offering a compelling alternative that may offer health benefits without the risks associated with live microorganisms [31,33,76,77]. Non-viable probiotics are also valuable in microbial therapy, especially for patients with compromised immune systems or in scenarios where introducing live bacteria poses risks. Evidence suggests that these compounds can influence gut microbiota composition and strengthen intestinal barrier function, potentially leading to significant clinical benefits [31,33].

Our findings demonstrate that the treatment with *OxxySlab* lysates effectively inhibits both the canonical and non-canonical TGF-β1 signaling pathways. It reduces TGF-β1 gene expression in activated myofibroblasts and reverses EMT features by restoring epithelial markers. However, these multifaceted antifibrotic effects have only been evaluated using in vitro models that can recreate a simple and controllable environment but are unable to mimic the complex structure of the human gut and the intricate process of intestinal fibrosis.

These results open the door to possible therapeutic applications for intestinal fibrosis in inflammatory bowel disease. While our current evidence pertains specifically to the bacterial lysate, it remains to be determined whether similar benefits may also arise from live probiotics. However, the precise microbial compounds responsible are not yet fully understood.

To elucidate the exact mechanisms of action of *OxxySlab* and its potential clinical applications, further mechanistic studies and experimentation using animal models are essential. In this regard, murine models are crucial for advancing our understanding of how both viable and non-viable probiotics function within the gut environment. Such models can help clarify mechanisms of action, determine optimal dosing strategies, and identify the specific conditions under which these probiotics exert their effects. Future investigations utilizing mouse models are anticipated to yield critical insights that can be translated into clinical practice, thereby guiding the development of effective probiotic therapies.

## 5. Conclusions

This study provides new insights into the antifibrotic potential of multi-strain probiotic formulations, highlighting mechanisms beyond microbiota modulation that may be therapeutically relevant in the context of IBD [78]. The ability of *OxxySlab* to interfere with key signaling pathways involved in intestinal fibrosis, such as proliferation, differentiation, and activation of fibroblasts and EMT, supports the use of probiotics as adjunctive agents in the management of IBD.

Ongoing advanced experimental models, including cell co-cultures, patient-derived organoids, and 3D full-thickness intestinal constructs, may further elucidate the dynamic mechanisms of fibrosis initiation and progression. The future goal is to identify the molecular switches that lead to fibrosis progression independent of the immune-inflammatory response and to explore how probiotics and their metabolites may prevent or reverse this transition.

## Figures and Tables

**Table 1:** List of the primary antibodies used for Western blot.

Primary Antibody	Dilution	Company
rabbit polyclonal anti-COL1A1	1:1000	Boster Biological Technology, Pleasanton, CA, USA
rabbit monoclonal anti-fibronectin	1:1000	Cell Signaling Technology, Danvers, MA, USA
rabbit monoclonal anti-p-SMAD2/SMAD3	1:1000	Cell Signaling Technology, Danvers, MA, USA
mouse monoclonal anti-α-SMA	1:1000	OriGene, Rockville, MD, USA
rabbit monoclonal Phospho-Akt (Ser473)	1:1000	Cell Signaling Technology, Danvers, MA, USA
rabbit polyclonal anti-Akt	1:1000	Cell Signaling Technology, Danvers, MA, USA
rabbit polyclonal anti-β-catenin	1:1000	Cell Signaling Technology, Danvers, MA, USA
rabbit monoclonal anti-PPAR-γ	1:1000	Cell Signaling Technology, Danvers, MA, USA
rabbit Polyclonal anti-occludin	1:1000	OriGene, Rockville, MD, USA
mouse monoclonal anti-E-cadherin	1:1000	Cell Signaling Technology, Danvers, MA, USA
mouse monoclonal anti-GAPDH	1:1000	OriGene, Rockville, MD, USA
mouse monoclonal anti-β-tubulin	1:1000	Thermo Fisher Scientific, Boston, MA, USA

**Table 2:** List of all the primary antibodies used for immunofluorescence.

Primary Antibody	Dilution	Company
rabbit polyclonal anti-COL1A1	1:250	Boster Biological Technology, Pleasanton, CA, USA
rabbit monoclonal anti-fibronectin	1:200	Cell Signaling Technology, Danvers, MA, USA
mouse monoclonal anti-α-SMA	1:250	OriGene, Rockville, MD, USA
rabbit Polyclonal anti-occludin	1:50	OriGene, Rockville, MD, USA
mouse monoclonal anti-E-cadherin	1:500	Cell Signaling Technology, Danvers, MA, USA

## Data Availability

The original contributions presented in this study are included in the article. Further inquiries can be directed to the corresponding author.

## Abbreviations

The following abbreviations are used in this manuscript:

ANOVA	Analysis of Variance
p-Akt	Phosphorylated-Akt
α-SMA	Alpha Smooth Muscle Actin
ATCC	American Type Culture Collection
BAs	Bile Acids
CCK-8	Cell Counting Kit-8
CD	Crohn’s Disease
CFU	Colony-Forming Unit
DMEM	Dulbecco’s Modified Eagle’s medium
ECM	Extracellular-Matrix
EMT	Epithelial–Mesenchymal Transition
GAPDH	Glyceraldehyde-3-Phosphate Dehydrogenase
IBDs	Inflammatory Bowel Diseases
IECs	Intestinal Epithelial Cells
LPS	Lipopolysaccharide
OD	Optical Density
PPAR-γ	Proliferator-Activated Receptor-gamma
p-Smad2/3	Phosphorylated-Small Mothers Against Decapentaplegic (SMAD) 2/3
RT-qPCR	Reverse Transcription-quantitative PCR
SCFAs	Short-Chain Fatty Acids
TGF-β1	Transforming Growth Factor β 1
UC	Ulcerative Colitis
