# Correction: Ciafarone et al. Multi-Strain Probiotic Lysate Attenuates TGF-β1-Induced Intestinal Fibrosis and EMT Modulating Smad, Akt, and WNT/β-Catenin Pathways. *Cells* 2025, *14*, 1432

**DOI:** 10.3390/cells15070599

**Published:** 2026-03-28

**Authors:** Alessia Ciafarone, Serena Artone, Valeria Ciummo, Francesca Rosaria Augello, Serena Altamura, Francesca Lombardi, Giovanni Latella, Paola Palumbo, Benedetta Cinque

**Affiliations:** 1 Department of Life, Health & Environmental Sciences, University of L’Aquila, 67100 L’Aquila, Italy; alessia.ciafarone@univaq.it (A.C.); serena.artone@univaq.it (S.A.); francescarosaria.augello@univaq.it (F.R.A.); serena.altamura@univaq.it (S.A.); francesca.lombardi@univaq.it (F.L.); paola.palumbo@univaq.it (P.P.); 2 Department of Innovative Technologies in Medicine and Dentistry, University “G. d’Annunzio”, 66100 Chieti, Italy; valeria.ciummo@graduate.univaq.it

**Error in Figure**

In the original publication [1], there was a mistake in Figure 6, panel F, image at the top left. For the DAPI staining image of the Control, the same image used for the TGFβ-treated cells was mistakenly inserted. We have now replaced it with the correct image. The corrected Figure 6 appears below. The authors state that the scientific conclusions are unaffected. This correction was approved by the Academic Editor. The original publication has also been updated.

## Figures and Tables

**Figure 6:**
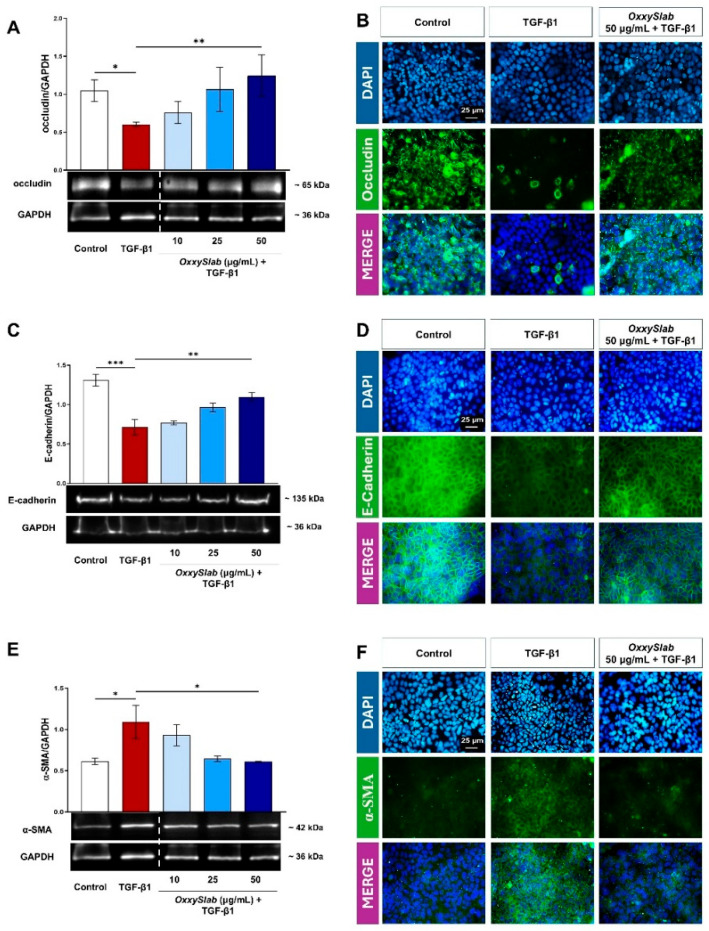
Effect of *OxxySlab* on epithelial and mesenchymal markers expression in the in vitro EMT model. Immunoblotting assay for (**A**) occludin and (**C**) E-cadherin, and (**E**) α-SMA was performed on Caco-2 IECs incubated for 96 h with TGF-β1 (20 ng/mL) alone or in combination with *OxxySlab* lysate at selected concentrations (10–25–50 µg protein/mL). Representative images of immunoblotting for occludin, E-cadherin, α-SMA, and GAPDH are shown. The dotted lines indicate areas in which the images were cropped to remove lanes irrelevant for this study. Densitometric analysis was normalized to GAPDH. Data are from three independent experiments, and values are expressed as mean ± SEM. Statistical analysis was performed using one-way ANOVA (* *p* < 0.05, ** *p* < 0.01, *** *p* < 0.001). No significant difference was detected between TGF and its combination with the lowest doses of probiotics. Representative immunofluorescence images from three independent experiments in duplicate, of untreated and treated Caco-2 IECs stained with antibodies against (**B**) occludin, (**D**) E-cadherin, (**F**) α-SMA antibodies (green), are shown. Nuclei were counterstained with DAPI (blue) (magnification 40×).
